# Anomalous origin of left circumflex artery from the right sinus of Valsalva: Clinical outcomes in a consecutive series of master athletes

**DOI:** 10.1002/clc.24120

**Published:** 2023-09-21

**Authors:** Angelo Ratti, Blanca Prestini, Edoardo Conte, Davide Marchetti, Matteo Schillaci, Eleonora Melotti, Marta Belmonte, Saima Mushtaq, Maria Antonietta Dessani, Francesca Pizzamiglio, Fabrizio Tundo, Paolo Zeppilli, Antonio Bartorelli, Daniele Andreini

**Affiliations:** ^1^ Department of Clinical Sciences and Community Health University of Milan Milan Italy; ^2^ Division of University Cardiology IRCCS Ospedale Galeazzi Sant'Ambrogio Milan Italy; ^3^ Cardiovascular Center Aalst OLV Clinic Aalst Belgium; ^4^ Department of Advanced Biomedical Sciences University Federico II Naples Italy; ^5^ Sport Cardiology Unit Centro Cardiologico Monzino, IRCCS Milan Italy; ^6^ Sports Medicine Unit Fondazione Policlinico Universitario Agostino Gemelli IRCCS Rome Italy; ^7^ Department of Biomedical and Clinical Sciences University of Milan Milan Italy

**Keywords:** anomalous origin of a coronary artery arising from the opposite sinus, anomalous origin of the left circumflex artery, master athletes, recreational athletes

## Abstract

**Background:**

Aim of the study was to collect and describe a case series of consecutive master athletes in whom an anomalous origin of left circumflex artery (LCx) from the right sinus of Valsalva (ALCx) was detected at a clinically indicated coronary computed tomography angiography CCTA) to establish a focused clinical management and counseling about sport activity in those subjects.

**Methods:**

We analyzed a prospective registry of subjects referred to a clinically indicated CCTA. Information about the clinical status was obtained by previous clinical records and clinical evaluation at time of image acquisition; follow‐up allowed to record symptoms, outcomes and downstream testing.

**Results:**

The study population consisted in 14 subjects, of which one competitive athlete and 13 recreational master athletes. Mean age was of 67.2 ± 10.6 years (71% of male); follow‐up lasted 6.4 ± 2.6 years. The major high‐risk anatomy features (inter‐arterial course, intramural segment, high take‐off and slit‐like ostium) were absent. None had abnormal ostial morphology and all had full retroaortic course; three subjects (21%) presented an acute take‐off angle. Coronary artery disease (CAD) was present in 10 patients (71%). Major outcomes (cardiac hospitalization, death for all causes) recorded were not related to the anomalous LCx. Symptoms were most related to atherosclerotic CAD in different vessels whereas two subjects without CAD exhibited cardiac symptoms, without hospitalization.

**Conclusions:**

Our study suggests that the diagnosis of ALCx, being usually associated to low‐risk anatomical characteristics, could be considered a benign finding, with scarce or no implications for physically active individuals neither for recreational athletes.

## Graphical abstract

Main findings of the study. In a series of master athletes, the anomalous left circumflex artery originating from the right sinus of Valsalva presented low‐risk anatomical characteristics at CCTA (absence of inter‐arterial course, intramural segment, high take‐off and slit‐like ostium) and was not associated with major clinical events.

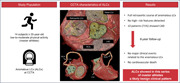

## 1 INTRODUCTION

Coronary arteries anomalies (CAAs) are rare congenital conditions with a wide range of clinical presentation, most commonly benign but in rare cases associated with life‐threatening cardiac events.

The estimated prevalence of CAAs is variable, depending mostly on detection mode and definition of normal variant; the known range of prevalence, based on invasive coronary angiography (ICA), coronary computed tomography angiography (CCTA), and autopsy databanks varies from 0.21% up to 5.79%. The anomalous origin of a coronary artery arising from the opposite sinus (ACAOS) of Valsalva represents a large part of CAAs^1^; its exact prevalence has recently been reported to be 0.8% in a population who underwent CCTA for clinical reason.^2^ Exercise‐related sudden cardiac death (SCD) risk is considered higher in case of anomalous left coronary artery origin because of the larger amount of myocardium at ischemic risk.^3^

Until the development of CCTA, which allowed for non‐invasive examination of the coronary arteries, ACAOS were mostly detected using ICA; screening is possible also by trans‐thoracic echocardiography,^1^, ^4^ despite patient anatomy and operator‐dependent variability, and cardiac magnetic resonance.^5^

The anomalous origin of the left circumflex artery (LCx) from the right sinus of Valsalva or from the right coronary artery (ALCx) is considered the most frequent CAA with an angiographic incidence of up to 0.67%.^6^, ^7^ After the anomalous take‐off, LCx usually shows a retroaortic course to reach the left atrio‐ventricular groove. This anomaly is usually considered benign,^1^ but only isolated case reports have been previously described.

Aim of the present study was to collect and describe a case series of consecutive physically active subjects (“master athletes”) in whom an ALCx was detected at a clinically indicated CCTA, in order to achieve more data on the right and safe clinical management and counseling about sport activity in those cases.

## 2 MATERIALS AND METHODS

### 2.1 Study population

We conducted a retrospective analysis from a prospective registry of subjects referred to the IRCCS Centro Cardiologico Monzino (Milan) who underwent CCTA for clinical reason between January 2007 and October 2015 and were diagnosed to have a CAA. Indications to perform the exam included: suspicion of coronary artery disease (CAD) because of anginal or non‐anginal chest pain and/or dyspnoea in patients at low to intermediate risk of CAD based on European Society of Cardiology (ESC) guidelines; follow‐up evaluation of previously known CAD; multiple cardiovascular risk factors and low‐to‐intermediate risk of CAD.

During the study period, 12 593 CCTA were performed and 92 patients with CAA were identified, thus with a prevalence of 0.71%. For the purpose of the study, only subjects with ALCx were selected (*n* = 18). As 4 patients have been lost to follow up, we focused this study on 14 patients. The clinical status of the subjects, at the time of CCTA, has been assessed through the information obtained either by previous clinical records and by the clinical evaluation performed at the time of image acquisition.

### 2.2 CCTA acquisition

Image acquisition was performed through a 64‐slice scanner (VCT, GE Medical System) with 640 × 625 mm collimation and 300‐ms gantry rotation time; tube voltage and tube current were matched to the patient's body mass index. Metoprolol was intravenously administered before CCTA scan in those subjects in which heart rate was more than 65 bpm. A bolus of 80 mL of high concentration contrast (Iomeron 400 mg/mL, Bracco Imaging) was administered intravenously at 5 mL/s, followed by 50 mL of saline injected at the same infusion rate. The bolus‐tracking approach was used to start the scan.

### 2.3 CCTA analysis

Patients were assessed for the presence of ALCx. The retrospective CT evaluation concerned qualitative and quantitative analysis of high‐risk anatomy features:

− Proximal vessel morphology: minimum and maximum diameters were measured at the most narrowed location and at the normal distal reference segment, used to categorize proximal vessel morphology as: (i) normal, (ii) “oval” (50%), and (iii) “slit‐like” narrowing (≥50% reduction in minimum diameter in the absence of CAD).
− Acute take‐off angle: the measured angle was between (a) the plane formed by the ostium center to a point 5 mm along the vessel centerline, and (b) a plane tangent to the aorta in multiplanar axial reconstruction at the level of the ACAOS ostium. Acute take‐off angle is defined as an angle showing a value of <45 degrees.
− Presence of intramural course: direct visualization of the vessel within the aortic wall (optimized by window width/level ≈1000/300), and the absence of adjacent epicardial fat (tissue region of interest mean signal 230 Hounsfield Units).
− Type of the proximal course of the anomalous vessel (interarterial, retroaortic, precardiac, subpulmonary).^2^, ^8^
− Vessel take‐off level: high take‐off was defined as >10 mm above sino‐tubular junction.^9^ This feature has importance for surgical planning.

Anomalous LCx were characterized by ostia type, that can be separate or shared; otherwise LCx can appear as a branch vessel.

All patients were evaluated for the presence of coronary atherosclerotic lesions, their location, characteristics (calcific, mix, soft) and the degree of stenosis. In this study, we focused on atherosclerotic lesions of the anomalous LCx.^2^, ^8^

### 2.4 Clinical analysis

The analysis concerned demographic data (age, gender), physical activity, cardiovascular risk factors and the presence of clinical symptoms (palpitation, non‐anginal chest pain, angina, dyspnea, syncope and heart failure) at the time of CT examination. The patient's medical histories were also evaluated retrospectively for the presence of arrhythmia and known CAD, associated or not with revascularization, either percutaneous coronary intervention (PCI) or coronary artery bypass graft, previous stress tests and results.

Subsequently, patients were contacted and asked if any symptoms, arrhythmias, cardiac events or death for all causes had occurred during the follow‐up. Cardiac events were defined as cardiac death, non‐fatal acute coronary syndrome, and any hospitalization for cardiovascular causes (revascularization for CAD progression, arrhythmia including electrical cardioversion or trans‐catheter ablation for atrial fibrillation, angina, exacerbation of heart failure). Moreover, patients were asked if they performed any cardiac stress test during the follow up period.

## 3 CASE SERIES DESCRIPTION

### 3.1 Population characteristics

Our case series consists of 14 patients with a mean age at diagnosis of 67.2 ± 10.6 years (range: 38–83 years), and a male prevalence of 71% (Table 1). As regards to cardiovascular risk factors, hypertension was the most prevalent (7 patients; 50%), followed by dyslipidemia (5 patients; 36%) and family history of CAD (4 patients; 29%); 3 patients were smokers and 1 had diabetes. Our study population was formed by one competitive athlete and 13 recreational athletes (master category). Following the current ESC guidelines for the definition of intensity of physical activity^10^ (based on the type and frequency), patients mostly performed low to moderate physical activity (7 low, 4 medium, and 3 high intensity physical activity, mainly long‐distance walking, running, cycling, and gym).

**Table 1:** Clinical data and CCTA characteristics of patients with anomalous origin of the circumflex artery from the right sinus of Valsalva (ALCx).

*N*	Age at CCTA exam	Sex	Sport	Baseline symptoms and arrhythmias	CAD risk factors	Known CAD and previous revascularization	CAD at time of CCTA	LCx atherosclerosis	Anatomical CCTA details^a^
1	38	F	Recreational	No	Family history	No	No	No	Shared ostium
2	83	M	Recreational	AF	No	no	Two‐vessels obstructive CAD	20% calcific proximal plaque	Shared ostium; acute take off angle
3	64	M	Recreational	No	Hypertension	No	No	No	Shared ostium
4	66	M	Recreational (cycling and gym)	No	Family history, dyslipidemia	One‐vessel obstructive CAD; PCI of LAD	Three‐vessels obstructive CAD	50% calcific proximal and mid plaques	Separate ostia
5	71	F	Recreational	No	Hypertension, dyslipidemia	Two‐vessels obstructive CAD (PCI of LCX and RCA)	Two‐vessels obstructive CAD	Previous PCI	Separate ostia
6	66	M	Recreational	Non anginal chest pain, AF	Dyslipidemia, smoking habit	No	No	No	Separate ostia
7	67	M	Recreational	No	Hypertension	No	One‐vessel non obstructive CAD	No	Separate ostia; acute take off angle
8	60	M	Recreational (cycling)	Palpitation, AF	No	No	One‐vessel obstructive CAD	No	Shared ostium; acute take off angle
9	76	M	Recreational	No	Family history, Hypertension, dyslipidemia, smoking habit, DM	No	Two‐vessels obstructive CAD	No	Shared ostium
10	70	M	Recreational	Non anginal chest pain	No	Two‐vessels obstructive CAD; PCI of LAD/D1	Two‐vessels obstructive CAD^6^	70% mixed distal plaque	Shared ostium
11	70	M	Recreational	No	Hypertension, dyslipidemia, Smoking habit	Non‐obstructive CAD	One‐vessel obstructive CAD	No	Shared ostium
12	68	M	Competitive (golf)	No	Family history, Hypertension	No	One‐vessel non obstructive CAD	No	Shared ostium
13	62	F	Recreational	AF	No	No	One‐vessel obstructive CAD	No	Separate ostia
14	80	F	Recreational (cycling, gym and swimming)	No	Hypertension	No	No	No	Shared ostium

Abbreviations: AF, atrial fibrillation; CAD, coronary artery disease; CCTA, coronary computed tomography angiography; D1, diagonal branch; DM, diabetes mellitus; LAD, left anterior descending artery; LCX, left circumflex artery; PCI, percutaneous coronary intervention; RCA, right coronary artery.

a In all cases, the anomalous LCx arose from the right sinus of Valsalva and had a full retroaortic course. There was no high take off and no intramural segment documented.

Subject #8 was symptomatic for palpitations, patients #6 and #10 for non‐anginal chest pain.

Among the 14 subjects enrolled, 4 patients (29%) had known CAD at time of evaluation, already treated by PCI in three cases (one of those, patient 5, by stenting of LCx), whereas atrial fibrillation had already been detected in 4 patients.

### 3.2 CCTA characteristics

All patients, as per inclusion criteria, had LCx arising from the right sinus of Valsalva (Table 1). Nine cases (64%) showed a shared ostium whereas in the remaining 5 (36%) subjects two separate ostia were evident. None had abnormal ostial morphology and all of them had full retroaortic course. An acute take‐off angle was documented in 3 patients (21%). The major high risk anatomy features (inter‐arterial course, intramural segment, high take‐off and slit‐like ostium), described for CAA but unusual for the anomalous LCx, were absent in our series. Atherosclerotic lesions were present in 10 patients (71%) and were obstructive (i.e., ≥50% stenosis) in at least one vessel in 9 cases. Four of those patients showed CAD involving LCx: patient #5 previously had LCx treated by PCI, patient #10 had significant stenosis (70%), patient #4 showed 50% stenosis and patient #2 only mild stenosis.

**Figure 1:**
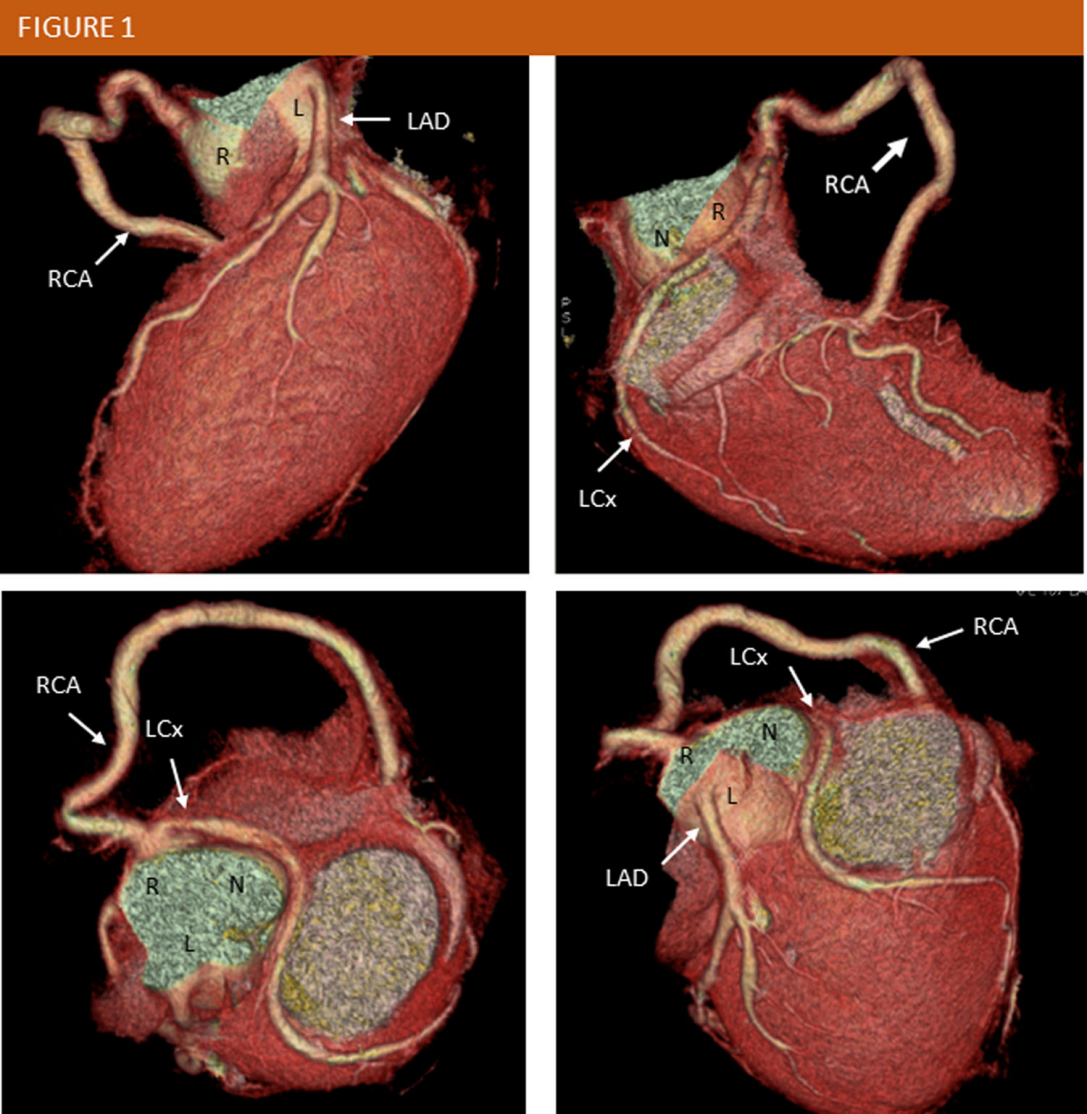
CCTA volume rendering images of anomalous origin of left circumflex artery from the right coronary sinus with shared ostia. L: left coronary sinus; N: non coronary sinus; R: right coronary sinus. CCTA, coronary computed tomography angiography; LAD, left anterior descending; LCx, left circumflex artery; RCA, right coronary artery.

**Figure 2:**
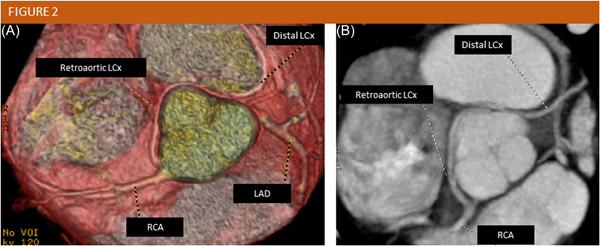
CCTA volume rendering (A) and maximum intensity projection (B) images showing a retro‐aortic left circumflex artery that arises from the right sinus of Valsalva. CCTA, coronary computed tomography angiography; LCx, left circumflex artery; RCA, right coronary artery.

Figures 1 and 2 show two case examples of anomalous LCx in one balanced‐dominance and one right dominant coronary system, respectively.

### 3.3 Clinical follow‐up

Mean duration of the follow‐up period in our study group was 6.4 ± 2.6 years (range: 2–12 years) (Table 2).

**Table 2:** Outcomes.

*N*	Follow up (years)	Symptoms and arrhythmias	Non‐fatal ACS	CV disease hospitalizations	PCI	Stress test	CV death	Death for all causes
1	8	Non‐anginal chest pain	No	No	No	Not performed	No	No
2	7	No	No	No	No	Not performed	No	No
3	3	Dyspnea; Ventricular extrasystole	No	No	No	Negative (ECG stress test)	No	No
4	12	No	No	Yes—TAVI (77y)	No	Borderline (cMR stress‐test)	No	No
5	9	No	Yes (72y)	Yes (72y)	PCI + DES D1 (72y)	Not performed	No	No
6	7	No	No	No	No	Not performed	No	No
7	4	No	No	No	No	Negative (ECG stress test)	No	No
8	5	No	No	Yes—TCA‐AF (61y)	No	Negative (ECG stress test)	No	No
9	7	Ventricular extrasystole	No	Yes—ICA (77y)	PCI + DES RCA (77y)	Not performed		Yes
10	8	No	No	Yes (72y)	PCI + DES LCx (70y)	Not performed	No	No
11	4	No	No	No	No	Not performed	No	No
12	7	No	No	No	No	Negative (ECG stress test)	No	No
13	2	Ventricular extrasystole	No	Yes—ICA (63y)	No	Not performed	No	No
14	6	No	No	Yes—HCM (81y)	No	Not performed	No	No

Abbreviations: CAD, coronary artery disease; cMR, cardiovascular magnetic resonance; CV, cardiovascular; DES, drug eluting stent; DM, diabetes mellitus; ECG, electrocardiogram; HCM, hypertrophic cardiomyopathy; ICA, invasive coronary angiography; LAD, left anterior descending artery; LCX, left circumflex artery; PCI, percutaneous coronary intervention; RCA, right coronary artery; TAVI, transcatheter aortic valve implantation; TCA‐AF, transcatheter ablation of atrial fibrillation.

No limitation to physical activity was recorded.

Follow‐up analysis showed seven cardiac events requiring hospitalization:

− Patient #10, at age of 70, was referred to ICA because of CCTA result; the procedure confirmed progression of CAD on the anomalous LCx, treated by PTCA and stenting.
− Patients #9 suffered progression of CAD, leading to planned revascularization (at age of 77), not related to the anomalous LCx.
− One non‐fatal acute coronary syndrome was reported (patient #5, at age of 72) and underwent percutaneous revascularization of the culprit lesion (a well‐represented diagonal branch).
− Patient #13, known for LAD obstructive CAD at CCTA, showed ventricular extrasystole, but ICA did not detect significant CAD.
− Patient #8 underwent ablation of AF at age of 61.
− Patient #4 underwent transcatheter aortic valve implantation at the age of 77.
− Patient #14 suffered HF due to her known HCM, requiring hospitalization.

Patient #3 showed ventricular extrasystole and patient #1 complained about non‐anginal chest pain, both without detection of obstructive CAD neither needing hospitalization.

Five patients performed stress test evaluation for clinical reason: one showed borderline result (patient #4, known for three‐vessels obstructive CAD, at cMR stress test); four electrocardiogram (ECG) stress tests were negative for myocardial ischemia (patients #3, #7, #8, and #12). Patient #12, the competitive golf player, was asymptomatic during the 7‐year follow‐up; he performed an ECG stress test, which resulted negative for inducible ischemia. No cardiovascular death was recorded; one non‐cardiac death was reported (patient #9).

## 4 DISCUSSION

The major findings of the present study may be summarized as following: (1) the isolated origin of LCx from the opposite sinus of Valsalva shows low‐risk anatomical characteristics at CCTA; (2) no major clinical events related to the anomalous LCx were observed in our study, suggesting its “benign” clinical pattern also in recreational athletes.

Practicing regular sport and training is common in the general population and being committed to official competition is not needed to engage high volumes of exercise; recreational athletes, as those in our case series, represent a not rare clinical profile who can face the diagnosis of CAA and need proper counseling and/or treatment. While rare, sudden death associated with sports is a disastrous event and several studies suggest that a CAA is between the main causes of exercise‐related SCD^10^, ^11^; this makes of utter importance to recognize which anomaly can be considered at risk, to start as soon as possible appropriate management (namely surgical therapy and/or sport restriction).

CCTA, in contrast to ICA, has some advantages in risk stratification of ACAOS, because it enables three‐dimensional analysis of the anatomy of both coronary arteries and of the whole heart, allowing for the evaluation of structures surrounding an abnormally arising and coursing coronary artery.^2^, ^12^ The main hypothesis about how ACAOS can be dangerous is the potential induction of ischemia, that can trigger infarction, arrhythmias, or sudden death. In those ACAOS with acute take‐off angle of the anomalous vessel, that makes the ostium slit‐like in shape, during increased cardiac output (e.g., exercise) the aorta dilates and the slit‐like ostium may become severely narrowed, leading to ischemia and its threatening cascade. Another potential mechanism of decreased coronary perfusion may be the compression of the anomalous vessel due to its passage between the aorta and pulmonary trunk.^13^, ^14^ Anatomical characteristics of the anomalous LCx in our patients were consistent with known previous data: LCx arising from the wrong sinus is not associated with high‐risk features,^1^, ^2^, ^15^ indeed in our population there was no inter‐arterial course, intramural segment, slit‐like ostium, or high take‐off. Moreover, during follow‐up no cardiovascular death occurred, underlining the good prognosis associated to this CAA. Of note, all patients presenting with symptoms at the time of CCTA or during follow‐up exhibited a different cardiological issue explaining them; no apparently healthy patient in the population presented any symptom at the time of diagnosis. Two patients developed symptoms during follow‐up without other known cardiac disease, one subject developing exertional chest pain and another exhibiting ventricular extrasystole. Although there is no certain clinical evidence on allocate these symptoms to the anomalous LCx, we can't exclude that some alteration to myocardial perfusion could be present in these cases, even if not significant enough to compromise clinical outcome. Being ischemia an imbalance between supply of oxygen (coronary blood flow) and demand (largely for contractile function), we underline that the concept of “high” workload in this context can't be absolute, because different individual characteristics (age, sex, training, type of exercise) change the workload that can be tolerated; this is an everyday‐life problem for patients, who need to know what kind of activity and what level of exercise they can perform without worrying for their health (and/or life). The answer, to date somehow uncertain, that the cardiologist can give is based on the risk profile of the individual CAA and the presence of ischemia during a stress test. With our case series, we show an anatomical low‐risk profile of LCx arising from the opposite sinus; moreover, between patients who underwent a stress test in our population only one tested positive for inducible ischemia, for reason likely unrelated to the anomalous LCx (known three‐vessels obstructive CAD). Two patients underwent revascularization during their life because of CAD on the anomalous LCx; however, their clinical profile was no different from many other CAD patients, making difficult with our data to assign a definite causative role to the LCx anomaly. We underline that all 4 patients with LCx obstructive CAD had also obstructive plaques involving at least another vessel, showing a general greater burden of atherosclerosis if LCx is involved. Our data is not sufficient to distinguish if this is due to patient's predisposition to atherosclerosis or to the presence of the anomaly; however, the largest single‐center study on adult patients with ACAOS to date available (793 patients),^16^ showed findings indicating that, compared to ACAOS of other coronaries, ALCx was associated with increased stenosis in not only the LCx itself but all the other coronaries. The authors proposed two possible reasons: (i) coronary hemodynamics, which could be altered in the anomalous LCx itself, due to the unusual length and retroaortic course, and in the overall coronary tree, given the smaller caliber of the anomalous LCx; (ii) timing of diagnosis with CCTA, considering that, possibly being the anomalous LCx a more benign variant, patients would become symptomatic at a later stage of coronary atherosclerosis, thus undergo CCTA and CAA diagnosis. Coronary hemodynamics play a pivotal role in CAD initiation and progression. In fact, it is known that localized pressure gradients, especially on a short segment of the artery, could alter the laminar flow normally present in the coronary artery producing areas subject to wall shear stress, which could lead to lipid accumulation, plaque progression and inflammation.^17^ Other prior studies proposed that, in case of anomalous LCx, these areas could be located at the junction point of the bound portion of the anomalous artery and the free portion as it wraps around the aorta.^18^, ^19^ Anyway, the main finding of the study showed that overall, the anomalous origin of LCx does not increase the atherosclerotic stenosis severity in the anomalous coronary; a limitation of the study was the comparison of CAD only within patients diagnosed with ACAOS and the lack of propensity‐matched patients without ACAOS as true controls. Other conflicting findings are available in literature: a study of 34 ACAOS patients, of which 19 LCx cases,^20^ found no increase in coronary stenosis in anomalous coronaries; Frescura et al.^21^ presented similar results in a case series of 27 patients with ACAOS. Meanwhile, studies by Click et al.^18^ and Samarendra et al.^19^ found increased CAD stenosis in the anomalous coronaries, typically limited to the anomalous LCx; despite this, clinical outcomes were not worse than matched controls. A more recent study^8^ showed that subjects with anomalous LCx origin show more common significant coronary atherosclerosis than those with other ACAOS, but the highest prevalence of chest pain and cardiac events were observed in the anomalous right coronary artery (ARCA) group; this was attributed by authors to the high‐risk anatomy features that are most common in patients with ARCA. These conflicting findings may be attributed to the limited sample sizes and to the uncertainty about the true prevalence of ACAOS; before some recommendation can be made about more intensive medical management or surveillance for CAD in patients with ALCx compared with the general population, more investigations are needed, clarifying true prevalence of those anomalies and their clinical outcomes.

### 4.1 Study limitations

In interpreting our findings, some limitations may be recognized: (1) Our study population is mostly formed by middle‐aged or elderly subjects, which are known to have a higher prevalence of CAD. This can make it difficult to understand the link between the CAA and acute coronary events. However, we were able to exclude that acute coronary events or elective coronary revascularization observed in our series were related to the myocardial territory covered by the anomalous LCx. (2) Our population consisted in recreational athletes and only one competitive athlete (who practiced a low intensity skills sport as golf); therefore, we can't generalize our findings to a different population of sportsmen in terms of younger age and higher exercise intensity.

## 5 CONCLUSIONS

Our data, providing imaging and clinical findings, suggests that the diagnosis of an isolated LCx arising from the wrong sinus of Valsalva, being usually associated to low‐risk anatomical characteristics, could be considered a benign finding, with scarce or no implications for physically active individuals neither for recreational athletes.

## CONFLICT OF INTEREST STATEMENT

The authors declare no conflicts of interest.

## Data Availability

The data that support the findings of this study are available on request from the corresponding author. The data are not publicly available due to privacy or ethical restrictions.
